# Supplementary material for: Long term temporal trends in synoptic-scale weather conditions favoring significant tornado occurrence over the central United States
Source: PLoS One. 2023 Feb 22;18(2):e0281312. doi: 10.1371/journal.pone.0281312 (PMC9946245; doi:10.1371/journal.pone.0281312)
Supplement: S1 File — (ZIP) [file pone.0281312.s001.zip › SupportingInfo.pdf]

## Supporting information

Here, we explain how to compute Bayesian posterior probabilities for each of the temporal trend models described in the methods section. We also explain how to use these posterior probabilities to compute the posterior probability of the existence of a temporal trend. Specifically, we consider BIC-based Bayesian posterior probabilities for the competing models [1, 2].

We assume a uniform prior across the three possible cases considered: no temporal trend ($M_0$), linear temporal trend ($M_1$), and temporal trend with slope breakpoint ($M_\theta$). That is, each of these cases receives a prior probability of one-third. Note that in the model $M_\theta$ with slope breakpoint, $\theta$ denotes the month when the breakpoint occurred. The possible values of $\theta$ are 1 through 456 and represent each month from January 1980 to December 2017. Further, we assume that a slope breakpoint may have occurred between January 1981 and December 2016. Specifically, we distribute the prior probability for the temporal trend with slope breakpoint uniformly across the possible breakpoint values $\theta = 13, \ldots, 444$. As a result, the prior probability of Model $M_{\theta^*}$, that is, the prior probability of a temporal trend with breakpoint at $\theta^*$ is $1/(3 \times 432)$.

Let $BIC(M_k)$ be the BIC for model $M_k$, where $k = 0, 1, \theta$. In addition, let $m_k(D) = e^{-0.5BIC(M_k)}$ be the BIC-based unnormalized marginal likelihood of model $M_k$. Hence, $m_0(D)$ is the marginal likelihood of no temporal trend, $m_1(D)$ is the marginal likelihood of a linear temporal trend, and $m_{\theta^*}(D)$ is the marginal likelihood of a temporal trend with slope breakpoint at $\theta = \theta^*$.

Then, by Bayes' Theorem [3, 4] the posterior probability of the linear trend model with a slope breakpoint at $\theta^*$ is

$$Pr(M_{\theta=\theta^*}|D) = \frac{\frac{1}{3\times 432} m_{\theta^*}(D)}{\frac{1}{3}m_0(D) + \frac{1}{3}m_1(D) + \frac{1}{3\times 432}\sum_{\theta^*=13}^{444} m_{\theta^*}(D)}, \tag{1}$$

and analogous formulas can be used to compute the posterior probability of $M_0$ (no trend) and of $M_1$ (linear trend).

Finally, the posterior probability (PP) of the existence of a temporal trend is equal to the sum of the posterior probabilities of a linear temporal trend and of a temporal trend with slope breakpoint, that is,

$$Pr(M_1|D) + \sum_{\theta^*=13}^{420} Pr(M_{\theta=\theta^*}|D). \tag{2}$$

More detailed information about the analysis using the temporal trend model with slope breakpoint may be obtained by examining the posterior probability of the breakpoint being at $\theta^*$, that is, $Pr(M_{\theta=\theta^*}|D)$. In particular, a point estimate of the breakpoint location is given by the posterior mode, that is, $\widehat{\theta} = \text{argmax}_\theta Pr(M_{\theta=\theta^*}|D)$.

## References


1. Kass RE, Raftery AE. Bayes Factors. J Am Stat Assoc. 1995;90(430):773. doi:10.2307/2291091.

2. Kass RE, Wasserman L. A Reference Bayesian Test for Nested Hypotheses and its Relationship to the Schwarz Criterion. J Am Stat Assoc. 1995;90(431):928–934. doi:10.1080/01621459.1995.10476592.

3. Robert C. The Bayesian Choice. 2nd ed. Springer Texts in Statistics. New York, NY: Springer New York; 2007. Available from: `https://doi.org/10.1007{%}2F0-387-71599-1http://link.springer.com/10.1007/0-387-71599-1`.

4. Gelman A, Carlin JB, Stern HS, Dunson DB, Vehtari A, Rubin DB. Bayesian Data Analysis, Third Edition. 3rd ed. Chapman and Hall/CRC; 2013.
