# Supplementary material for: Long term temporal trends in synoptic-scale weather conditions favoring significant tornado occurrence over the central United States
Source: PLoS One. 2023 Feb 22;18(2):e0281312. doi: 10.1371/journal.pone.0281312 (PMC9946245; doi:10.1371/journal.pone.0281312)
Supplement: Attachment — Submitted filename: Response to Reviewers.docx [file pone.0281312.s002.docx]

# Editor Letter

PONE-D-22-28676

Long term temporal trends in synoptic-scale weather conditions favoring significant tornado occurrence over the central United States

PLOS ONE

Dear Dr. Zick,

Thank you for submitting your manuscript to PLOS ONE. After careful consideration, we feel that it has merit but does not fully meet PLOS ONE’s publication criteria as it currently stands. Therefore, we invite you to submit a revised version of the manuscript that fully addresses all the points raised during the review process by both reviewers.
Please submit your revised manuscript by Jan 06 2023 11:59PM. If you will need more time than this to complete your revisions, please reply to this message or contact the journal office at [plosone@plos.org](mailto:plosone@plos.org). When you're ready to submit your revision, log on to <https://www.editorialmanager.com/pone/> and select the 'Submissions Needing Revision' folder to locate your manuscript file.

Please include the following items when submitting your revised manuscript:

- A rebuttal letter that responds to each point raised by the academic editor and reviewer(s). You should upload this letter as a separate file labeled 'Response to Reviewers'.
- A marked-up copy of your manuscript that highlights changes made to the original version. You should upload this as a separate file labeled 'Revised Manuscript with Track Changes'.
- An unmarked version of your revised paper without tracked changes. You should upload this as a separate file labeled 'Manuscript'.

If you would like to make changes to your financial disclosure, please include your updated statement in your cover letter. Guidelines for resubmitting your figure files are available below the reviewer comments at the end of this letter.

If applicable, we recommend that you deposit your laboratory protocols in [protocols.io](http://protocols.io/) to enhance the reproducibility of your results. Protocols.io assigns your protocol its own identifier (DOI) so that it can be cited independently in the future. For instructions see: <https://journals.plos.org/plosone/s/submission-guidelines#loc-laboratory-protocols>. Additionally, PLOS ONE offers an option for publishing peer-reviewed Lab Protocol articles, which describe protocols hosted on [protocols.io](http://protocols.io/). Read more information on sharing protocols at <https://plos.org/protocols?utm_medium=editorial-email&utm_source=authorletters&utm_campaign=protocols>.

We look forward to receiving your revised manuscript.

Kind regards,

João Miguel Dias, Ph.D.
Academic Editor
PLOS ONE

Dear João Miguel Dias, Ph.D.,

Thank you for your recommendations regarding our manuscript, " Long term temporal trends in synoptic-scale weather conditions favoring significant tornado occurrence over the central United States."

We have carefully considered the reviewer's comments, and the revised manuscript addresses their recommendations.

Best Regards,

Mohamed El Khouly, Stephanie Zick, and Marco Ferreira

# Journal Requirements:

When submitting your revision, we need you to address these additional requirements.

1. Please ensure that your manuscript meets PLOS ONE's style requirements, including those for file naming. The PLOS ONE style templates can be found at 
<https://journals.plos.org/plosone/s/file?id=wjVg/PLOSOne_formatting_sample_main_body.pdf> 
and 
<https://journals.plos.org/plosone/s/file?id=ba62/PLOSOne_formatting_sample_title_authors_affiliations.pdf>.

Following the PLOS One instructions, we used [latexdiff](https://www.ctan.org/pkg/latexdiff?lang=en) to generate a difference file showing the revisions to the original manuscript. Unfortunately, there were numerous fatal errors in that diff.tex file, and we had to manually edit it so that the tex file could be compiled. We looked over the manually edited file very carefully, and we believe that the associated PDF reflects the changes accurately. The errors were mostly related to changes that we made to the tables (in response to reviewer #1’s comment about the number of decimal digits).

We also followed the instructions here (<https://journals.plos.org/plosone/s/latex#loc-references>) to include the references directly in the .tex file.

Lastly, we made a slight change to the title page so that the corresponding author is more clearly labeled.

If any other formatting edits are needed, please let us know.

2. Please note that PLOS ONE has specific guidelines on code sharing for submissions in which author-generated code underpins the findings in the manuscript. In these cases, all author-generated code must be made available without restrictions upon publication of the work. Please review our guidelines at <https://journals.plos.org/plosone/s/materials-and-software-sharing#loc-sharing-code> and ensure that your code is shared in a way that follows best practice and facilitates reproducibility and reuse.

We have created a data repository with the Virginia Tech Libraries. The repository includes all software and data that is needed to follow the research in this manuscript. Here is the data citation:

El Khouly, Mohamed; Zick, Stephanie; Ferreira, Marco (2022): Software and Data for "Long term temporal trends in synoptic-scale weather conditions favoring significant tornado occurrence over the central United States". University Libraries, Virginia Tech. Dataset. <https://doi.org/10.7294/21750689>

3. We note that Figure 1 in your submission contain [map/satellite] images which may be copyrighted. All PLOS content is published under the Creative Commons Attribution License (CC BY 4.0), which means that the manuscript, images, and Supporting Information files will be freely available online, and any third party is permitted to access, download, copy, distribute, and use these materials in any way, even commercially, with proper attribution. For these reasons, we cannot publish previously copyrighted maps or satellite images created using proprietary data, such as Google software (Google Maps, Street View, and Earth). For more information, see our copyright guidelines: <http://journals.plos.org/plosone/s/licenses-and-copyright>.
 
We require you to either (1) present written permission from the copyright holder to publish these figures specifically under the CC BY 4.0 license, or (2) remove the figures from your submission:
 
a) You may seek permission from the original copyright holder of Figure 1 to publish the content specifically under the CC BY 4.0 license.  
 
We recommend that you contact the original copyright holder with the Content Permission Form (<http://journals.plos.org/plosone/s/file?id=7c09/content-permission-form.pdf>) and the following text:
“I request permission for the open-access journal PLOS ONE to publish XXX under the Creative Commons Attribution License (CCAL) CC BY 4.0 (<http://creativecommons.org/licenses/by/4.0/>). Please be aware that this license allows unrestricted use and distribution, even commercially, by third parties. Please reply and provide explicit written permission to publish XXX under a CC BY license and complete the attached form.”
 
Please upload the completed Content Permission Form or other proof of granted permissions as an "Other" file with your submission.
 
In the figure caption of the copyrighted figure, please include the following text: “Reprinted from [ref] under a CC BY license, with permission from [name of publisher], original copyright [original copyright year].”
 
b) If you are unable to obtain permission from the original copyright holder to publish these figures under the CC BY 4.0 license or if the copyright holder’s requirements are incompatible with the CC BY 4.0 license, please either i) remove the figure or ii) supply a replacement figure that complies with the CC BY 4.0 license. Please check copyright information on all replacement figures and update the figure caption with source information. If applicable, please specify in the figure caption text when a figure is similar but not identical to the original image and is therefore for illustrative purposes only.
The following resources for replacing copyrighted map figures may be helpful:
 
USGS National Map Viewer (public domain): <http://viewer.nationalmap.gov/viewer/>
The Gateway to Astronaut Photography of Earth (public domain): <http://eol.jsc.nasa.gov/sseop/clickmap/>
Maps at the CIA (public domain): <https://www.cia.gov/library/publications/the-world-factbook/index.html> and <https://www.cia.gov/library/publications/cia-maps-publications/index.html>
NASA Earth Observatory (public domain): <http://earthobservatory.nasa.gov/>
Landsat: <http://landsat.visibleearth.nasa.gov/>
USGS EROS (Earth Resources Observatory and Science (EROS) Center) (public domain): [http://eros.usgs.gov/#](http://eros.usgs.gov/)
Natural Earth (public domain): <http://www.naturalearthdata.com/>

Thank you for this comment. We have completely regenerated Figure 1 using the open-source R package ggmap. Therefore, there is no longer a need to obtain a license.

# Reviewers' comments:

## Reviewer #1:

General Comments

The manuscript “Long term temporal trends in synoptic-scale weather conditions favoring significant tornado occurrence over the central United States” (PONE-D-22-28676), submitted to PLOS ONE, deals with a relevant topic, it is generally well written and overall, it is interesting for a wide audience. However, some aspects are not clear and a number of minor, mostly formal corrections including typos and figure improvements, are needed. I would like to see it published but I do not think it should be accepted in its current form. Therefore I recommend a review following as guidance the items listed below.

We thank the reviewer for their helpful guidance towards improving the research and the clarity of the findings throughout the manuscript.

Specific Comments

1. Page 2, last paragraph. When listing the variables considered (air temperature, relative humidity, etc.) it is not clear if specific levels or all levels of the MERRA-2 reanalysis data are considered.

We provide this information in section 2 (Materials and Methods) so we’ve added a reference to that section to the text here.

2. Page 3, line 69. The text mentions ‘section 2’ here, but sections are not numbered. Perhaps, ‘the second section’ or something similar would be more appropriate.

Done

3. Page 8, Table 4. For a given variable (column), please check if the same number of decimal digits are needed.

In Table 4, we have generally included 3 decimal digits. For smaller coefficients, we have included four decimal digits. The corresponding standard errors have the same number of decimal digits as their coefficients. We have adjusted a few of the results in Table 4 so that all decimal digits meet the standards outlined above. For example, for the Intercept for R1, the standard error was 2.95, and we have change it to 2.950. Tables 5-6 have also been adjusted accordingly.

4. Page 11, line 332 (and 339 and other references similarly). Reference ‘Nielsen-Gammon and Gold’ is not numbered (as other references given in the text) – please check journal style citation format.

Thank you for catching this. We’ve added the Nielsen Gammon numbered citation here.

5. Page 11, line 341. Please correct typo: Figure ?? c-d.

Done

6. Page 12, line 362. ‘Atmospheric Instability Mode’: if there is a ‘Moist Static Stability Mode’ should include the term ‘Dry’ or something similar, to avoid possible misunderstandings?

Thank you for this comment. We’ve decided to rename this mode to Dry Static Stability Mode since that also describes this EOF mode but with more specificity, and also distinguishes it from the MSS mode.

7. Page 12, Table 6. Despite 6 EOF modes are described in Results section b) only 4 are listed in this table. Perhaps I missed something but I think this should be clarified (maybe instead of using the term ‘noteworthy EOF modes’ in the table, you could simply call them ‘Selected EOF modes’ or something similar; alternatively, a larger table listing all 6 modes could be considered).

Table 6 only includes the modes with significant temporal changes. We have updated the table caption to better explain why those modes were included, and we’ve updated the text to refer to these modes as “selected EOF modes.”

8. Page 13, line 409. Similarly, as item 4, reference unnumbered.

Done

9. Page 14, lines 464-465. The no inclusion of EF0 cases affects previous results? If so, I think this should be explained earlier, when describing the datasets used. Otherwise, please clarify in text.

As noted in this section, there was a a jump in their recorded numbers after the introduction of Doppler radar (Agee and Childs 2014). Including EF0 tornadoes would likely lead to the identification of spurious trends; therefore, we have excluded these tornadoes from the present study. We have added another sentence in this section to clarify the reason for excluding the EF0 tornadoes.

10. Page 15, line 498. Typo: please add blank space 6,R1 -> 6, R1

Done

11. Page 16, Conclusion sections indicates that ‘5 synoptic-scale ingredients’ were identified – from the 6 EOF modes found (but only 4 selected to be shown in Table 6). I found this a bit confusing; perhaps authors could try to reformulate this explanation linking it to the previous discussion.

Thank you for this note. We do want to avoid confusion. Here, we refer to 5 modes that have been previously identified in the research literature. Then, there is an additional novel (STF) mode that has not been previously identified. We’ve done some rewording in this section to make this distinction clearer.

12. Page 17, last paragraph. Please make sure that all locations mentioned in the text (Illinois, Indiana, etc.) appear labeled in Figure 1 so readers not familiar with the area of study can follow the explanation.

Done. Thank you for this suggestion.

13. Page 21. Please check Ref. 16 and 24 are apparently duplicated.

Thank you for catching this! Fixed.

14. Page 23. Ref. 45: please rewrite the article title (now it is in upper case).

Done

15. Page 23. Ref. 50, typo: andtornado -> and tornado

Done

16. Page 24. Ref. 68, typo: unbalaced -> unbalanced ?

Done

17. Page 25. Ref. 78: please rewrite authors names (now they are in upper case).

Done

18. Please check figures resolution and quality (labels of fig. 2, 3, 5 and 6, etc.).

We have reformatted most of the figures, including adding larger text for most figure labels. We hope this helps with readability.

## Reviewer #2:

Overview: The authors present a well-written and timely manuscript on an important topic. I find no major issues with the data or analyses, and have only minor suggestions (mainly for the intro and conclusion).

We thank the reviewer for their encouraging comments and for suggestions to improve the literature review and overall context of our findings in the conclusions section.

Introduction: Incomplete representation of recent relevant literature (details below).

Line 7: Authors use reference 4 to support their claim of negative and positive trends in plains and eastern US. This study is built on numerous studies that came before it. Citing numerous studies from the body of literature from 2014-present would be stronger and would account for the numerous methods that have been used to illustrate these opposing trends.

Thank you for this suggestion. We have added four additional references to support this claim.

Line 8-10: Authors state that the increase in the eastern US is mainly explained by increases in population density. I disagree with this statement. This reference shows that population density combined with radar explain less than 30% of the variability.

We have reworded this sentence to say “partially explained.”

Line 9: Should this say "while decreases in the US Plains..."?

The wording is (partially) correct but needs refining. There was an increase in detected tornadoes until the early 2000s because of improved radar technology. These are those tornadoes that move through the prairies/grasslands (nowhere near people) and were never detected before. More recently, numbers have declined, as you note. Therefore, we reworded this sentence to instead note that “variability” in the US Plains region is partially explained by advancements in radar technology.

Line 28-30: The literature does illustrate regional variability in the trends of proxies, but there is agreement across studies in the regional variability (important point that I think is missing in this characterization).

Thank you. This is a good point. We have added an additional sentence to the paragraph: “Importantly, that regional variability is generally consistent across studies, with numerous findings that the large-scale environment in the southeastern U.S. is becoming more favorable for severe weather.”

Line 341: Should this reference be to Figure 3?

Fixed. Thank you!

Line 343: Please cite the studies that this result is similar to.

Thank you, this is a good suggestion. We’ve added a few references to studies that define the dynamic tropospause this way.

Line 559-563: Is the STF mode missing in this list?

Thank you for this note. Here, we refer to 5 modes that have been previously identified in the research literature. Then, there is an additional novel (STF) mode that has not been previously identified. We’ve done some rewording in this section to make this distinction clearer.

Line 587-589: It would be insightful to discuss these [STF] trends in the context of documented regional tornado trends.

Great idea. Thank you for this suggestion. We have added a sentence about the implications of an increased STF mode in this region and how this might affect tornado frequency, and we’ve cited the relevant studies as well.
